# Advanced Epithelioid Malignant Peripheral Nerve Sheath Tumor Showing Complete Response to Combined Surgery and Chemotherapy: A Case Report

**DOI:** 10.1155/2011/705345

**Published:** 2011-09-06

**Authors:** Tomohiro Minagawa, Ryuta Shioya, Chigusa Sato, Ryuji Shichinohe, Go Yasui, Kohsuke Ishikawa, Hiroko Takahashi

**Affiliations:** ^1^Department of Plastic and Reconstructive Surgery, Asahikawa-Kosei General Hospital, 1 Jo-dohri 24-chome 111, Asahikawa City, Hokkaido 078-8211, Japan; ^2^Department of Plastic and Reconstructive Surgery, Hokkaido University Graduate School of Medicine, Sapporo, Hokkaido 060-0815, Japan

## Abstract

Malignant peripheral nerve sheath tumor (MPNST) is a rare high-grade soft tissue sarcoma. The epithelioid variant accounts for 5% or less of MPNSTs; the clinical behavior of this variant is unclear. Reports of approximately 40 cases are available in the English literature; however, most reports addressed clinicopathological features rather than therapeutic procedures or clinical courses. We describe a case of a 62-year-old male with an epithelioid MPNST of the left foot. Multiple lung metastases developed after radical surgery on the primary lesion. The response to adjuvant chemotherapy including doxorubicin and ifosfamide was favorable, and thoracoscopic resection was subsequently performed on the remaining three metastases. No evidence of recurrence or metastasis was observed at the 12-month followup after the first operation. Further followup and chemotherapy may be required.

## 1. Introduction

Malignant peripheral nerve sheath tumor (MPNST) is a rare malignant soft tissue tumor. Its variants include rhabdomyoblastic, glandular, and epithelioid MPNST [1]. Epithelioid MPNST (EMPNST) accounts for 5% or less of MPNSTs [1]. Approximately 40 cases of EMPNST have been reported in the English literature [2–5]. Most reports address clinicopathological features; however, few reports are available on advanced EMPNST with precise treatment procedures. Here, we report an additional case of EMPNST arising in the foot and involving lung metastases that showed complete response to combined surgery and chemotherapy.

## 2. Case Presentation

**Figure 1:**
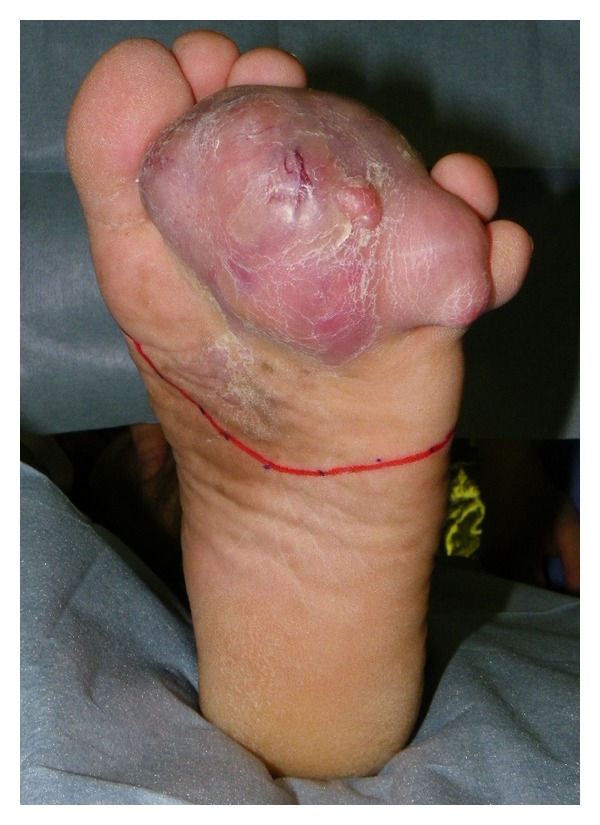
Preoperative view showing an enlarging mass in the third web space.

**Figure 2:**
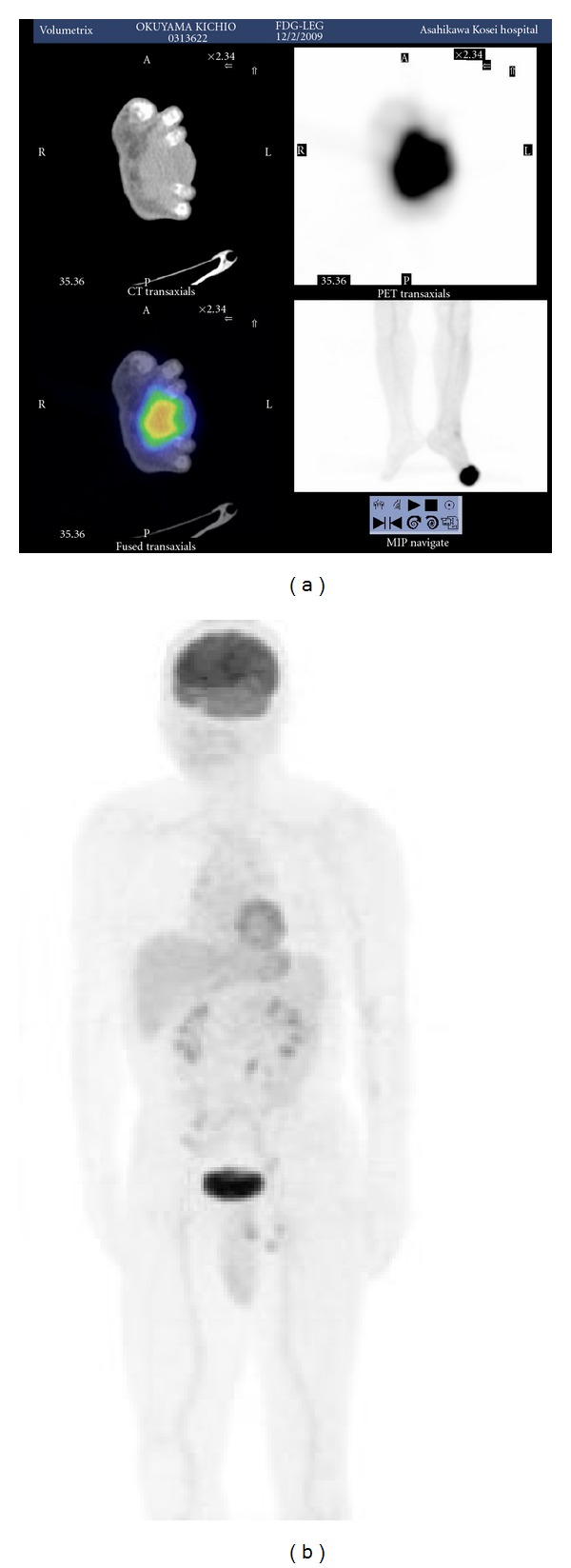
Preoperative positron emission tomography. (a) Abnormal uptake involving the affected foot. (b) No abnormal uptake was observed except left inguinal region.

A 62-year-old male without neurofibromatosis presented with an enlarging, painful mass in the third web space of his left foot. The lesion was first noticed approximately five years earlier as a nodular lesion that was then diagnosed as an epidermal cyst of the sole at a dermatologic clinic. Neither excision nor biopsy was performed at that time. On examination, there was a firm, round mass measuring 9 × 7 cm in the third web space (Figure 1). The mass was immobile against the surrounding bones and adhered to the overlying skin. Multiple swollen lymph nodes were palpable in the left inguinal region. An open biopsy was performed, which showed proliferation of epithelioid round cells with cellular atypia, indicating probable malignant melanoma. Radiological examination including fluorodeoxyglucose positron emission tomography showed no evidence of definite distant metastases except left inguinal region (Figures 2(a) and 2(b)). Therefore, surgical treatment was planned under the diagnosis of T4aN2bM0, stage III-C malignant melanoma according to the American Joint Committee on Cancer classification.

**Figure 3:**
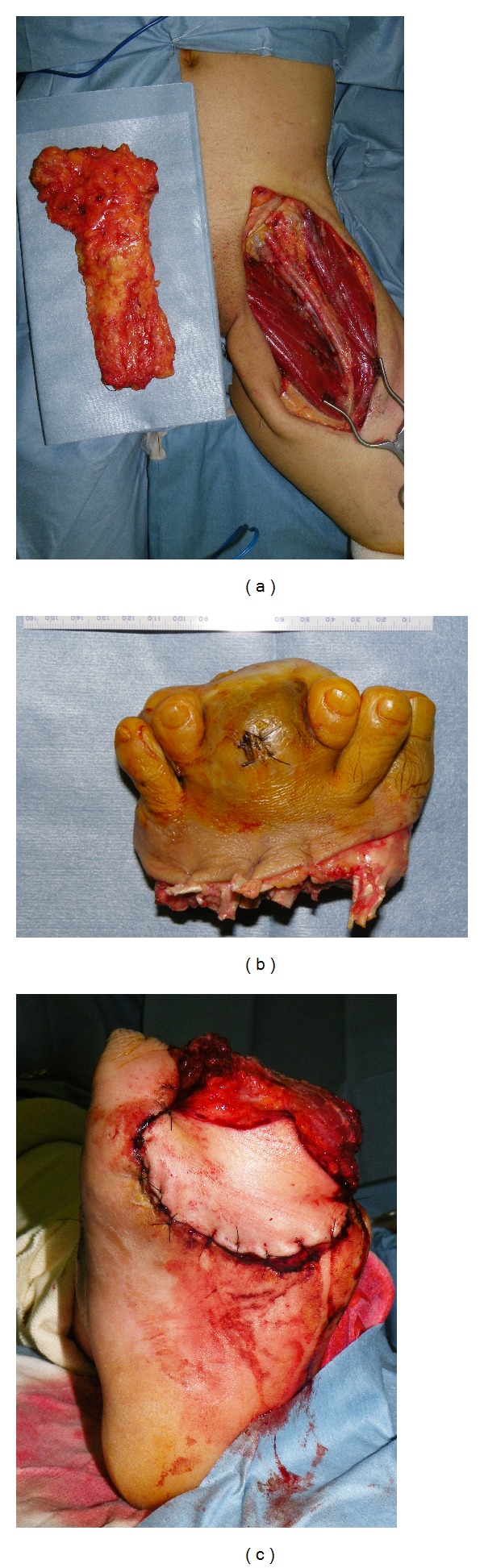
Intraoperative views of ablative surgery and secondary reconstructive surgery. (a) Ipsilateral inguinal dissection at the first operation. (b) Wide excision of the primary lesion with a 30 mm surgical margin. (c) Free latissimus dorsi musculocutaneous flap transfer to the amputated stump.

**Figure 4:**
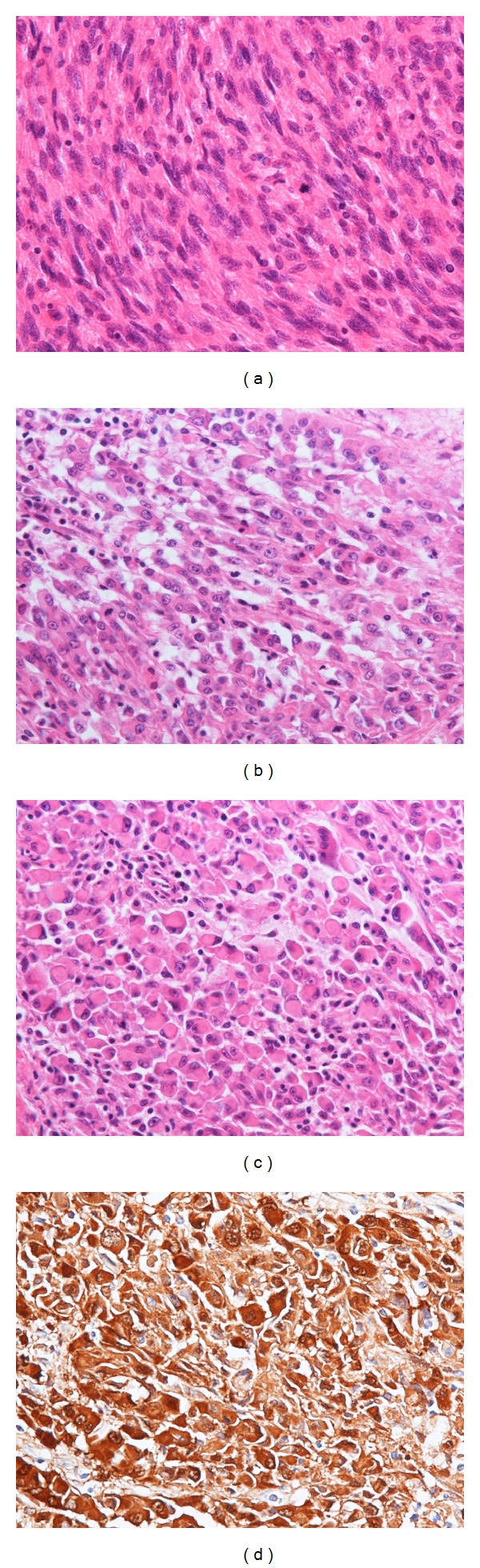
Histological findings. (a) Proliferation of spindle cells with abnormal mitotic activities reminiscent of conventional malignant schwannoma (hematoxylin-eosin, ×400). (b), (c) Epithelioid features of round-to-polygonal or rhabdoid cells with cellular atypia resembling malignant melanoma (hematoxylin-eosin, ×400). (d) Immunohistochemical staining demonstrating strongly positive for S-100 protein.

The first operation was performed under general anesthesia. Left inguinal dissection was carried out (Figure 3(a)), and an adequately wide excision of the primary tumor with a 30 mm surgical margin (i.e., transmetatarsal amputation) was performed (Figure 3(b)). The resultant raw surface of the stump was temporarily covered with an artificial dermis. One week postoperatively, the stump was resurfaced with a free latissimus dorsi musculocutaneous flap with a split-thickness skin graft following histological confirmation of adequate surgical margins (Figure 3(c)). Although partial necrosis of the flap was observed postoperatively, application of an additional split-thickness skin graft under local anesthesia led to complete wound healing. The specimen showed nodular growth of the lesion measuring 70 × 43 mm between the dermis and subcutaneous layer without continuity to the epidermis. Histologically, the lesion consisted of two components: proliferation of spindle cells with some differentiation to nerve cells, reminiscent of conventional malignant schwannoma (Figure 4(a)), and epithelioid proliferation of round-to-polygonal or rhabdoid cells with hyperchromatic nuclei, reminiscent of malignant melanoma (Figures 4(b) and 4(c)). Tumor cells stained positive for S-100 protein and negative for HMB45 (Figure 4(d)). All dissected lymph nodes were free of sarcoma. Based on these histological features, the lesion was diagnosed as EMPNST, of which histological grade was classified as grade 3 according to the FNCLCC system (tumor differentiation: score 3, mitotic count: score 2, and tumor necrosis: score 1).

**Figure 5:**
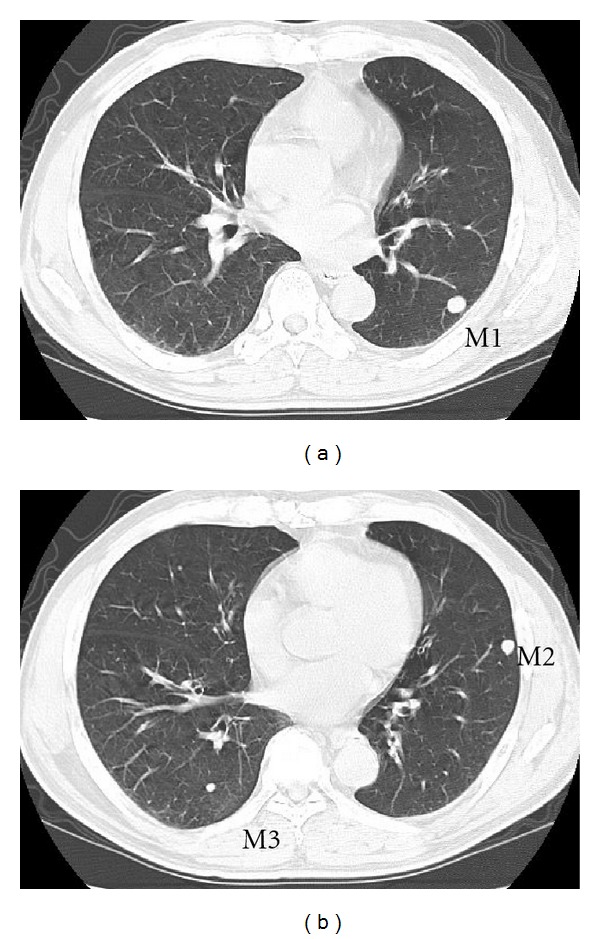
Ten weeks after the first operation. (a), (b) Development of multiple lung metastases (M1, M2, M3).

**Figure 6:**
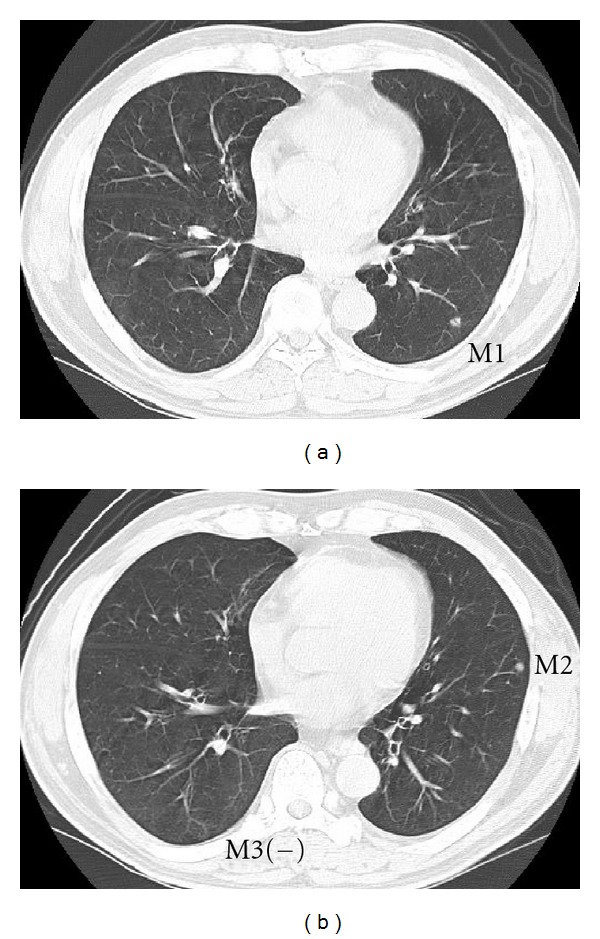
After two courses of adjuvant chemotherapy showing good response to all metastases. Note M3 was not recognizable.

**Figure 7:**
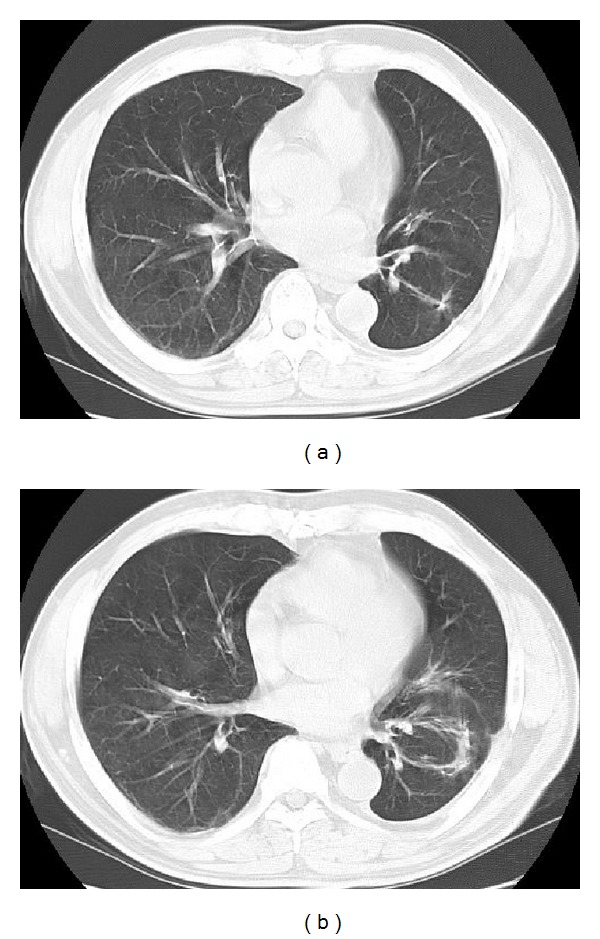
Twelve months after the first operation. All metastatic lesions were eliminated through the chemotherapy and thoracic surgery.

**Figure 8:**
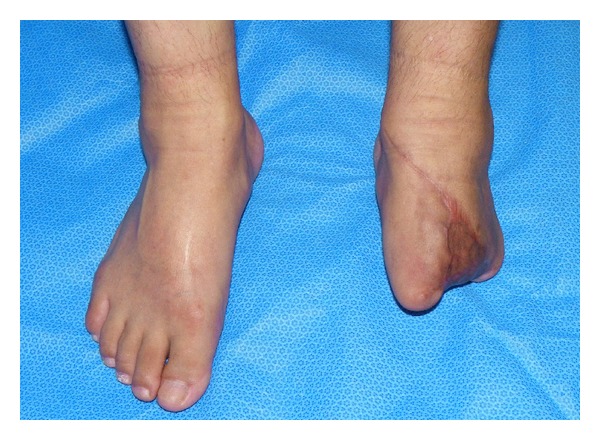
Clinical view at 12 month postoperatively.

 Ten weeks after the first operation, computed tomography demonstrated the development of multiple small bilateral lung nodules (Figures 5(a) and 5(b)). No further evidence of metastasis to other organs was observed. Systemic adjuvant chemotherapy consisting of doxorubicin and ifosfamide was then initiated. Doxorubicin (60 mg/m^2^) and ifosfamide (7.5 g/m^2^) with mesna (Uromitexan) were administered. After two courses of the adjuvant therapy, follow-up computed tomography revealed a good response to all abnormal shadows; however, two lesions in segment 8 and one in segment 10 of the left lung field were still observable (Figures 6(a) and 6(b)). Because these three lesions were located just beneath the pleura, tumors were resected during thoracoscopic surgery five months after the first operation. Histological examination revealed complete necrosis of the tumor cells in one lesion and metastases of EMPNST in the other two lesions. At the 12-month followup after radical surgery of the primary tumor, during which time four courses of adjuvant chemotherapy and a thoracic surgery were added, no signs of local recurrence or metastasis were observed (Figures 7(a) and 7(b)). The patient maintained ambulation without a brace (Figure 8).

## 3. Discussion

Most MPNSTs are generally considered high-grade sarcomas [1]. Approximately 40% of patients developed local recurrence, and the overall five-year survival rate was 34–43% [6, 7]. On the other hand, epithelioid variants are rare and are estimated to comprise 5% or fewer of MPNSTs [1]. Thus, biologic behavior and prognosis are unclear. However, the largest series, which included 26 cases and was reported by Laskin et al. [3], showed that although most patients were treated with wide excision, four developed distant metastases and three died of the disease within three years. High incidence of metastasis (seven of 14 cases) was also reported by Lodding et al. in 1986 [2].

 Aggressive surgery is considered the only procedure to improve prognosis of the MPNST [6, 7], and the response to chemotherapy against MPNST is poor according to previous reports [8]; however, several reports have demonstrated that chemotherapy can be effective [9, 10]. Although the standard regimens are still investigative, ifosfamide and doxorubicin are considered key drugs for advanced soft tissue sarcomas [11, 12]. In our clinical case, administering ifosfamide and doxorubicin with mesna showed good efficacy on the multiple lung metastases, with low-grade toxicities including myelosuppression and hair loss.

Surgical management of lung metastases may be effective in select situations [13]. Two reports on MPNST demonstrated poor survival rates after development of pulmonary metastases; however, prolonged survived cases with thoracotomy were also described [7, 8]. As for nonosteogenic sarcoma, Creagan showed the overall 5-year survival following the first thoracotomy was 29% with a median survival of 18 months [14]. The present case involved the development of disseminating lung nodules ten weeks postoperatively; however, chemotherapy provided a favorable response to all metastatic lesions. Subsequently, thoracoscopic resection of the remaining three lesions was performed to both eliminate the sarcoma and allow histological evaluation of the response to chemotherapy. Based on the histological findings that two of the three lesions showed remaining sarcoma, continuing the same regimen without surgery may risk inducing development of chemotherapy-resistant sarcoma cells.

## 4. Conclusions

An extremely rare case of advanced EMPNST was reported. Multiple metastases to both lungs developed despite the adequately wide excision of the primary tumor. However, adjuvant chemotherapy and thoracoscopic resection yielded a favorable and complete response at the 12-month followup.

##  Conflict of Interests

The authors declare that they have no competing interests.
